# From Knowledge to Habits: Changes in COVID‐19 Health Attitudes, Practices, and Sources of Information (HAPS) at Qatar University Post‐Pandemic

**DOI:** 10.1002/hsr2.70725

**Published:** 2025-06-04

**Authors:** Ibrahim Alkaabi, Magdy Abita, Amr Ouda, Yousif Mahdi, Mohammed Imad Malki

**Affiliations:** ^1^ College of Arts and Sciences, Deanship of General Studies Qatar University Doha Qatar; ^2^ Social Work Program, Department of Social Science, College of Arts and Sciences Qatar University Doha Qatar; ^3^ Department of Basic Medical Science, College of Medicine, QU Health Qatar University Doha Qatar; ^4^ Family Medicine Residency Program, Department of Medical Education Hamad Medical Corporation Doha Qatar; ^5^ Psycology Program, Department of Social Science, College of Arts and Sciences Qatar University Doha Qatar

**Keywords:** COVID‐19, habits, health, knowledge, pandemic measures, post pandemic, practices, sources of information

## Abstract

**Background and Aims:**

The COVID‐19 pandemic has profoundly impacted health behaviors globally and this study investigated changes in Health, Attitudes, Practices, and Sources of information (HAPS) among 1186 students, faculty, and staff at Qatar University following the lifting of pandemic restrictions. We aimed to identify demographic influences on HAPS and assess behavioral shifts from peak pandemic practices.

**Methods:**

A cross‐sectional survey adapted from the KAPS model, comprising 20 true/false questions, was administered to 1186 participants. The survey assessed demographics, health habits, public practices, attitudes towards COVID‐19, and information sources. Data was analyzed using IBM SPSS 28 and Excel.

**Results:**

Results revealed significant shifts: mask‐wearing decreased by 78% (from 90.5% to 10.7%, *p* < 0.001), and hand sanitizer use decreased by 35.6% (from 72% to 36.4%, *p* < 0.001). Handwashing, however, decreased only by 13.6% (from 69.9% to 56.3%, *p* < 0.001). Notably, older adults (50+ ; *n* = 69) reported the highest median HAPS scores. Based on publicly available university data, approximately 60% of this group are likely faculty or staff, with an estimated 75% being non‐Qatari, often from South Asian or other Arab countries. Non‐Qataris (*n* = 496) generally exhibited higher health habit (*p* = 0.015) and attitude scores (*p* < 0.001) compared to Qataris (*n* = 690). Social media was the primary source of COVID‐19 information (66.4%).

**Conclusions:**

This study reveals significant shifts in health behaviors and information sources among Qatar University's population post‐pandemic. These findings underscore the need for targeted interventions, particularly among younger and Qatari populations, to promote sustained healthy habits. Such interventions should leverage social media and address demographic disparities to improve pandemic preparedness. Understanding these changes is crucial for developing tailored public health strategies and improving pandemic preparedness.

## 1 Introduction

The COVID‐19 pandemic has had wide‐ranging effects on various aspects of daily life, encompassing health, the economy, education, and societal norms. Governments worldwide have implemented diverse policies and measures to contain the virus and safeguard their populations. These measures include the closure of nonessential businesses, the implementation of social distancing protocols, the enforcement of travel restrictions, and the cancellation of large gatherings. Despite the negative consequences of the pandemic, it has been argued that this approach has yielded certain benefits in preparing for future outbreaks. The policies developed and lessons learned during this pandemic are expected to contribute to improved preparedness plans, enhanced healthcare systems, and increased public awareness regarding the importance of hygiene and preventive measures for mitigating future outbreaks. Consequently, practices such as hand hygiene, social distancing, and the use of face masks have become more widely adopted and accepted than ever before [1, 2, 3].

To quantitatively assess these advancements, researchers have examined the Knowledge, Attitude, and Practices (KAP) as well as the Knowledge, Attitude, Practices, and Sources of information (KAPS) scores of populations worldwide in relation to the pandemic [4, 5, 6, 7]. Policymakers have utilized these data to identify areas of weakness and promote targeted awareness campaigns during the peak of the pandemic. Governments have implemented effective and targeted measures to safeguard their citizens from the virus, contributing to containment efforts and saving lives. The data‐driven approach to policymaking has been widely adopted because of its effectiveness in combating the pandemic [8]. In Qatar, COVID‐19 legal restrictions have been lifted. This change may lead the population to relax their adherence to health habits, which were initially followed more strictly due to fear of legal repercussions. By comparing scores before and after the lifting of restrictions, it becomes possible to assess which health habits and practices have been strengthened or weakened and to determine the true impact of the pandemic on a population.

Behavioral scientists and community medicine experts collaborated to create the Health, Attitudes, Practices, and Sources of Information (HAPS) model, advancing beyond the existing Knowledge, Attitudes, and Practices Survey (KAPS) model. The HAPS model's primary innovation lies in its emphasis on discerning participants' habits rather than solely assessing their knowledge levels. The rationale behind this shift is rooted in the recognition that awareness does not always translate into action, and the HAPS model seeks to quantify real‐life applications rather than knowledge alone. While building on the established frameworks of KAP and KAPS, the HAPS model introduces a nuanced approach to comprehensively understand health‐related behaviors in a population.

Through the analysis of the data, a deeper understanding of the long‐term impact of the pandemic can be gained. This information can then be used to develop more effective and targeted pandemic policies and to formulate improved public health campaigns and educational initiatives. Ultimately, this approach can aid decision‐makers in the optimal allocation of resources. As a follow‐up study, the present research aimed to assess the present HAPS of Qatari residents following the lifting of COVID‐19 restrictions and compare them with the KAPS scores of Qatar University attendees during the second wave of the pandemic. By examining the collective response of the university community to COVID‐19, this study offers insights into the broader community's response. A comparison will be made to a baseline study conducted during the peak of the COVID‐19 outbreak in Qatar titled “Knowledge, Attitude, Practices, and Sources of Information (KAPS) toward COVID‐19 during the second wave pandemic among the university population in Qatar: A cross‐sectional study” [9]. This comparative cross‐sectional analysis will provide valuable insights into evolving health behaviors and information sources post‐pandemic.

## 2 Materials and Methods

The survey was administered via a questionnaire consisting of 20 true or false questions relating to COVID‐19 health habits, practices, attitudes, information sources. Furthermore, demographic information such as sex, age, nationality, education, and occupation were collected as part of the questionnaire (refer to Supporting Information S1: Table S1). By analyzing these data, we aimed to better understand COVID‐19 related behaviors and identify potential gaps in reaching specific demographic groups. The survey instrument was adapted from a previously validated Knowledge, Attitudes, Practices, and Sources of Information (KAPS) tool used in a study conducted during the second wave of the pandemic (reference the previous study). This adaptation involved the following changes: (1) Four knowledge‐specific questions about COVID‐19 symptoms and transmission (e.g., “The incubation period of COVID‐19 is 2–14 days”) were removed, as the present study focused on post‐pandemic habits and behaviors rather than knowledge recall. (2) Six practice‐related questions (e.g., mask‐wearing, hand hygiene) were rephrased to assess current habits (e.g., “Do you still wear a facemask when in public at all times?”). (3) Four new questions were added to specifically address the long‐term impact of the pandemic on health habits, such as changes in diet, exercise, and smoking cessation intentions. The new questions were developed based on emerging literature on post‐pandemic behavioral changes. (4) The language was adjusted to suit the local Qatari context after consultation with local experts in public health and social sciences. This adaptation ensured alignment with the post‐pandemic context and the research aim of assessing behavioral changes from knowledge and awareness to sustained habits.

Participants were recruited via email invitations sent through the university's official email portal to all students, faculty, and staff (approximately 27,000 individuals) from January to April 2023. The email contained a brief explanation of the study's purpose and a link to the online survey. Participation was voluntary and anonymous. A reminder email was sent 2 weeks after the initial invitation to maximize the response rate. To further incentivize participation, respondents were offered the chance to enter a raffle for a small prize. This convenience sampling method may limit the generalizability of the findings to populations beyond the QU community; future research will explore more representative sampling strategies, such as stratified random sampling based on faculty, staff, and student proportions.

As part of the university's official email portal, students, faculty, and employees were emailed the questionnaire from January to April 2023. Responses were collected using a Yes/No/Sometimes format for the 20 true/false questions and checkbox questions for the information sources. For analysis, “Sometimes” responses were coded as “No” to create a binary variable (Yes = 1, No/Sometimes = 0). While this simplification was necessary for the current analysis, it reduced the potential for nuanced responses. This decision was made because “Sometimes” could indicate inconsistent behavior, and we wanted to focus on consistent adoption of healthy habits. However, we acknowledge that this might have led to an underestimation of certain practices. Future research will utilize Likert scales to capture a wider range of attitudes and behaviors. We will explore options for a 5‐point Likert scale (e.g., Always, Often, Sometimes, Rarely, Never) to allow participants to express the frequency or intensity of their actions more accurately.

A quantitative analysis of these data was performed using SPSS and Excel. This study was conducted in accordance with the ethical principles outlined in the Declaration of Helsinki. Ethical approval for this study was obtained from the Institutional Review Board (IRB) at Qatar University (IRB approval number: 1802‐E/23). All participants provided informed consent electronically before commencing the survey. They were informed about the purpose of the study, the voluntary nature of their participation, the confidentiality of their responses, and their right to withdraw from the study at any time without consequences.

### 2.1 Participants

As a community, Qatar University is home to nationals, residents, and international visitors from across the world with a wide variety of backgrounds, specialties, sociodemographic backgrounds, ages, and employment statuses. This study was conducted at QU, a university primarily comprised of undergraduates and postgraduates. Male and female participants were both welcome to participate.

To participate, students, staff, or faculty members must be older than the age of 18 and enrolled at Qatar University. The survey was conducted in English only. As a final decision, the research team excluded residents who moved to Qatar after February 2020 to prevent information bias arising from new residents. The survey was anonymous and voluntary, and participants were informed that their responses would not affect their academic or work status. All the responses were kept confidential, and the data were securely stored in accordance with Qatar University's data privacy policies. Participants with incomplete responses were excluded from the analysis to ensure data integrity for the primary analysis

The inclusion criteria were as follows:

- Aged > 18.
- Currently, a student, staff member, or faculty member at Qatar University.
- Able to read English.
- Has been in Qatar since February 2020.

The study's exclusion criteria were as follows:

- The subject does not consent.
- The subject does not complete all the questions.

The sample size for this study was calculated using a systematic approach based on principles of power analysis. The calculation was conducted to ensure the study's ability to detect meaningful effects with a high probability, thereby enhancing the robustness and reliability of the research findings. The optimal sample size for this study was calculated using the Creative Research Systems (CRS) calculator with a confidence level of 99% and a margin of error of 5%, with a sample size of 624 subjects needed to achieve statistical power.

The HAPS tool was developed with the collaboration of behavioral scientists and community medicine experts at Qatar University and was based on the existing tested and verified KAPS tool [9]. The modified survey was then piloted with a small group of 20 participants, representative of our target population, to assess clarity and face validity. Feedback from the pilot study led to minor wording adjustments to improve clarity and ensure cultural appropriateness. To complete the survey, which was only sent in English, participants had to devote approximately 8 min. The study was divided into five parts, each of which assessed a different aspect of the research. A box was first checked to capture general demographic data, including sex, age, nationality, education level, and employment status. Then, the participants were asked to rate their agreement with specific statements regarding the study topic. The tool was designed to be easily understood and was tested to ensure that the questions were unambiguous. Participants were asked to submit their responses at the end of the survey.

Using the Yes/No/Sometimes format, each of our four research questions addressed a specific component of the research question. In the second part, five questions were designed to assess participants' specific health habits, which were thought to have been developed during local awareness campaigns. Part three examined public practices, such as using hand sanitizers, cleaning one's hands for 20 s, wearing a face mask in public, avoiding gatherings altogether, keeping an appropriate distance from others, and attending social gatherings, regardless of whether the gathering took place indoors or outdoors. Part four involved assessing the attitudes of the population toward COVID‐19. As part of the questionnaire, participants were asked whether they reported suspicious symptoms of themselves, colleagues, friends, or even family members to the government and whether they were using the national case tracking app (Ehteraz). The research team also examined whether Qatar University's internal regulations for COVID‐19 were followed. HAPS scores were then calculated with higher scores indicating more positive health habits, attitudes, and practices. After successfully incorporating public sources into KAP in the last paper, the team decided to do the same. Participants were asked to indicate whether they relied on the following sources for information. News channels, government press conferences, social media, friends, relatives, and colleagues were all listed.

### 2.2 Statistical Analysis

Data were analyzed using IBM SPSS 28 and Excel. Descriptive statistics (frequencies, percentages, medians, interquartile ranges) were used to summarize demographic characteristics and HAPS scores. Nonparametric tests (Mann–Whitney U tests for two‐group comparisons and Kruskal–Wallis tests for multiple‐group comparisons) were employed to assess differences in HAPS scores across demographic groups due to the non‐normal distribution of the data, as indicated by significant results in both the Kolmogorov–Smirnov and Shapiro–Wilk tests for Practice, Attitude, and Boosting scores (*p* < 0.05). The use of nonparametric tests ensures robustness against deviations from a normal distribution, providing a more appropriate statistical approach given the observed data distribution. IBM SPSS 28 was used for statistical analyses. The comparison model used Microsoft Excel. *χ* ^2^ tests were used to compare the distribution of demographic variables between the KAPS and HAPS samples, as shown in Supporting Information S1: Table S2. To assess the relationship between attitude, health habits, demographics, and COVID‐19 practices, a multiple linear regression analysis was conducted with practice scores as the dependent variable and attitude scores, health habit scores, sex, nationality, and occupation as independent variables.

To address the potential impact of unequal sample sizes between the current HAPS study (*N* = 1186) and the previous KAPS study (*N* = 475), effect sizes (Phi coefficients) were calculated to quantify the magnitude of observed differences. The significance level was set at *p* < 0.05. The results were significant if the *p‐*value was less than 0.05. The results of the tests are presented in tables and figures.

### 2.3 Reliability Assessment

An internal consistency assessment of the questionnaire's key items was conducted through a pilot study with 20 participants. The pilot study participants were recruited from the same population as the main study but were not included in the final sample. The results of the pilot study indicated that the questionnaire was reliable and valid. The internal consistency of the questionnaire was further improved after the data was collected. The Cronbach's alpha increased from 0.706 to 0.714.

### 2.4 Comparison of Results From the Current HAPS Model to Those From the Previous KAPS Model

To ensure the validity of our comparisons between the current HAPS study (post‐pandemic) and the previous KAPS study (peak pandemic), we assessed the demographic composition of both samples. Despite the HAPS sample (*N* = 1186) being approximately three times larger than the KAPS sample (*N* = 475), *χ* ^2^ tests revealed no significant differences in the distribution of age, sex, nationality, educational level, or occupation (see Supporting Information S1: Table S2). This demographic equivalence strengthens the comparability of our findings across the two time periods.

For direct comparison of specific responses over time, we paired KAPS survey questions with corresponding HAPS questions, as detailed in Table 1. The rationale for using two‐tailed *z*‐tests to compare proportions stemmed from the following considerations:

- Large sample sizes: Both the KAPS (*N* = 475) and HAPS (*N* = 1186) samples are relatively large. The central limit theorem suggests that with large sample sizes, the sampling distribution of a proportion will approximate a normal distribution, even if the underlying data is not perfectly normal. This makes the *z*‐test a valid choice for comparing proportions.
- Independence of samples: The KAPS and HAPS surveys were conducted at different time points with different participants, ensuring the independence of the samples.
- Practical considerations: While other tests (e.g., *χ* ^2^ test with continuity correction) could be considered, the *z*‐test is a widely accepted and straightforward method for comparing proportions with large sample sizes. We believe it provides a valid and interpretable assessment of the differences in proportions between the KAPS and HAPS surveys.

**Table 1:** Mapping of KAPS and HAPS survey questions for comparative analysis of health behaviors and information sources.

KAPS component	KAPS question	HAPS component	HAPS question	Rationale for *z*‐test comparison
Knowledge	K1. The incubation period of COVID‐19 is 2–14 days.	N/A		Not applicable; HAPS focuses on habits, not knowledge.
	K2. The most common symptoms are fever, dry cough, and tiredness.	N/A		Not applicable; HAPS focuses on habits, not knowledge.
	K3. Older people and those with underlying conditions are at higher risk.	N/A		Not applicable; HAPS focuses on habits, not knowledge.
	K4. Supportive care is the current treatment for COVID‐19.	N/A		Not applicable; HAPS focuses on habits, not knowledge.
	K5. Having a healthy diet is essential for maintaining the immune system.	Health habits	Do you still eat healthy food to boost immunity?	Direct comparison of behavior before and after restrictions.
	K6. Smokers are not affected differently by COVID‐19.	Health habits	Do you still consider quitting smoking/passive smoking to boost your immunity?	Direct comparison of behavior before and after restrictions.
	K7. Using supplements is beneficial in preventing COVID‐19.	Health habits	Do you still take vitamins to boost immunity?	Direct comparison of behavior before and after restrictions.
	K8. Exercise helps reduce the impact of COVID‐19 symptoms.	Health habits	Do you still exercise regularly to boost immunity?	Direct comparison of behavior before and after restrictions.
Practice	P1. Have you attended large social gatherings?	Practice	Do you still attend large social gatherings indoors and outdoors?	Direct comparison of behavior before and after restrictions.
	P2. Do you socially distance by at least 1.5 m?	Practice	Do you still socially distance by at least 1.5 m from another person?	Direct comparison of behavior before and after restrictions.
	P3. Do you avoid crowded gatherings?	Practice	Do you still avoid crowded gatherings indoors and outdoors?	Direct comparison of behavior before and after restrictions.
	P4. Do you wear a facemask in public?	Practice	Do you still wear a facemask when in public at all times?	Direct comparison of behavior before and after restrictions.
	P5. Do you wash your hands with soap for 20 s?	Practice	Do you still wash your hands with soap and water for at least 20 s?	Direct comparison of behavior before and after restrictions.
	P6. Do you use hand sanitizer often?	Practice	Do you still use hand sanitizer?	Direct comparison of behavior before and after restrictions.
Attitude towards laws	A1. Do you use the Ehteraz application?	N/A		Not included in HAPS analysis as COVID‐19 laws were lifted during the post‐pandemic period.
	A2. Do you report suspicious symptoms?	N/A		Not included in HAPS analysis as COVID‐19 laws were lifted during the post‐pandemic period.
	A3. Do you follow QU instructions regarding COVID‐19?	N/A		Not included in HAPS analysis as COVID‐19 laws were lifted during the post‐pandemic period.
Resources	R1. News channels	Information sources	News channels	Assesses the same information source.
	R2. Government press conferences	Information sources	Government press conferences	Assesses the same information source.
	R3. Social media	Information sources	Social media	Assesses the same information source.
	R4. Family, relatives, friends, and coworkers	Information sources	Family, relatives, friends, and coworkers	Assesses the same information source.

However, we acknowledge that the collapsing of response categories might have resulted in some loss of information. Additionally, the unequal sample sizes may have led to reduced statistical power for detecting small differences in the KAPS sample. To address this, we have reported effect sizes (Phi coefficients) alongside *p*‐values. These effect sizes quantify the magnitude of the observed differences and provide a more comprehensive understanding of the practical significance of our findings, even in cases where statistical significance might not be reached due to limited power in the smaller sample.

Additionally, the “Attitude towards Laws” component was present in the KAPS survey, reflecting the presence of COVID‐19 regulations during the peak pandemic. This component was not included in the HAPS survey as these laws had been lifted by the time of the post‐pandemic assessment.

Therefore, we focused our comparisons on the following areas:

- Post‐pandemic health habits versus peak pandemic knowledge: We grouped questions from the KAPS knowledge section that pertained to health behaviors (e.g., healthy diet, smoking cessation) and compared them to the corresponding health habit questions in HAPS.
- Post‐pandemic practices versus peak pandemic practices: We directly compared the paired practice questions from both surveys.

Comparisons were made using two‐tailed *z*‐tests, calculating standard errors, *z*‐values, and *p*‐values to assess the statistical significance of any observed differences. The results of these comparisons are presented in Table 3.

## 3 Results

### 3.1 Demographic Information

**Figure 1:**
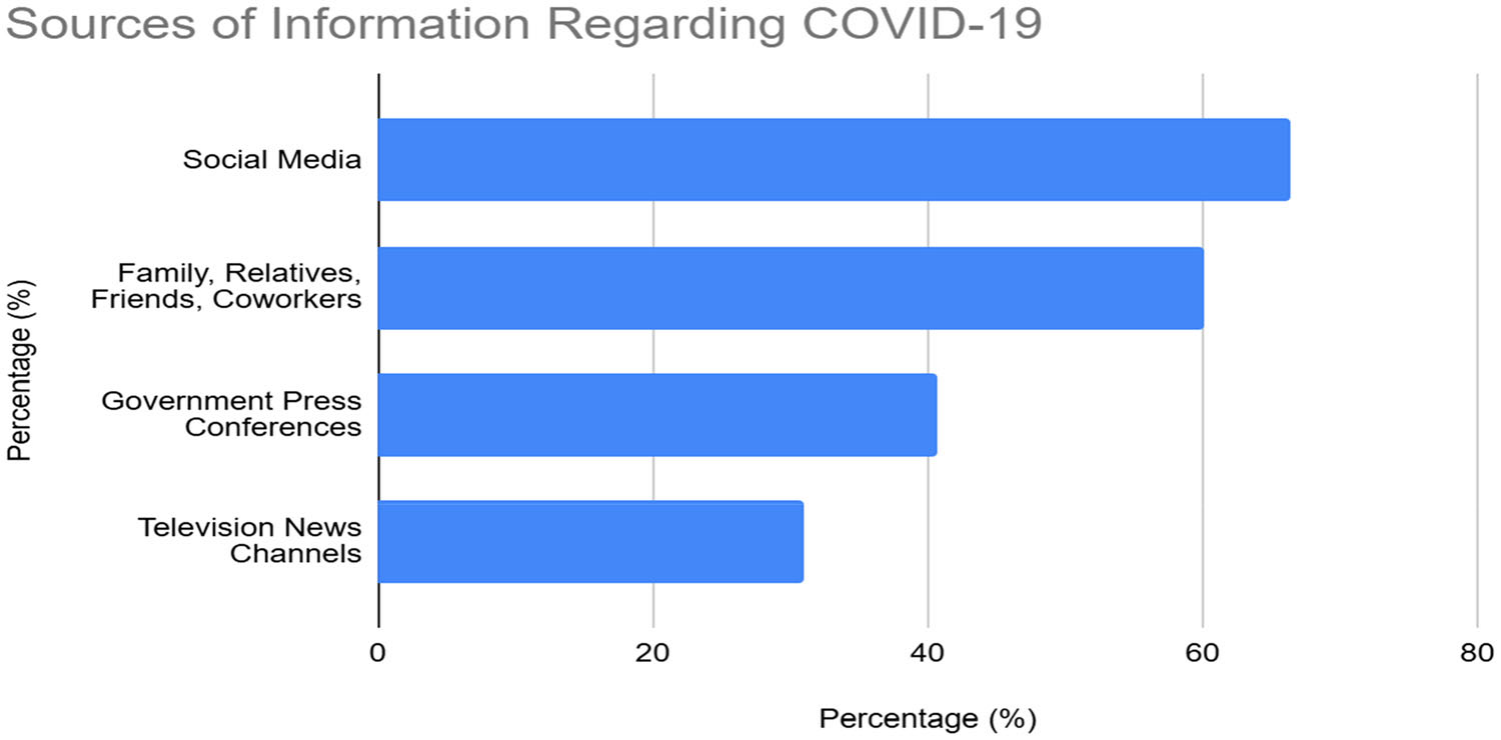
Primary sources of COVID‐19 information among Qatar university attendees. *Note:* Participants could select multiple sources.

This study included 1186 participants: 355 (29.9%) males and 831 (70.1%) females. The majority (53.1%) were aged 19–29. Most participants were Qatari (58.2%), held undergraduate degrees (80.3%), and were full‐time students (66.9%). Detailed demographic information is presented in Table 2 and Figure 1.

**Table 2:** Demographic characteristics of the participants.

	Characteristics	*N*	%
Gender	Male	355	29.9
	Female	831	70.1
Age	18 and below	298	25.1
	19–29	630	53.1
	30–39	98	8.3
	40–49	91	7.7
	50 and above	69	5.8
Nationality	Qatari	690	58.2
	Non‐Qatari	496	41.8
Education	Undergraduate	952	80.3
	Postgraduate	234	19.7
Occupation	Employed	393	33.1
	Full‐time students	793	66.9

### 3.2 HAPS Scores, Demographic Comparisons, Multiple Linear Regression, and Information Sources

We assessed differences in health habits, attitudes toward COVID‐19 guidelines, adherence to preventive practices, and changes in practices and behaviors from peak pandemic to post‐pandemic across various demographic groups (sex, nationality, education level, occupation, and age). The influence of these factors on COVID‐19 practices was further examined using multiple linear regression.

Table 3 summarizes the median HAPS scores, mean ranks, test statistics, *p*‐values, effect sizes, and interpretations for the demographic comparisons. Table 3 presents the results of the *z*‐tests comparing health practices and behaviors between the peak pandemic and post‐pandemic periods, along with effect sizes. Table 4 displays the multiple linear regression results predicting COVID‐19 practices from attitudes, health habits, and demographic factors. The specific details of these statistical comparisons, including median scores, mean ranks, and effect sizes, are presented in Table 3.

**Table 3:** Comparison of HAPS scores by demographic characteristics in Qatar university participants.

HAPS component	Characteristic	Group 1	Group 2	Mean (SE) group 1	Mean (SE) group 2	Median (IQR) group 1	Median (IQR) group 2	Test statistic	*p*‐value	Effect size	Interpretation
Health habits	Gender	Male	Female	2.61 (0.071)	2.33 (0.046)	4 (3, 5)	3 (2, 4)	*U* = 129,633	< 0.001	*r* = −0.15	Males had significantly higher scores (small‐to‐medium effect).
	Nationality	Qatari	Non‐Qatari	2.33 (0.052)	2.52 (0.057)	3 (2, 4)	3 (2, 5)	*U* = 157,347	0.015	*r* = −0.07	Non‐Qataris had significantly higher scores (small effect).
	Education	Undergraduate	Postgraduate	2.33 (0.043)	2.76 (0.080)	3 (2, 4)	4 (3, 5)	*U* = 90,015	< 0.001	*r* = −0.21	Postgraduates had significantly higher scores (small‐to‐medium effect).
	Occupation	Full‐time students	Employed	2.32 (0.046)	2.60 (0.069)	3 (2, 4)	4 (3, 5)	*U* = 137,494	< 0.001	*r* = −0.15	Employed individuals had significantly higher scores (small‐to‐medium effect).
	Age	18 and below	19–29	2.23 (0.076)	2.38 (0.054)	3 (2, 4)	3 (2, 4)				
		30–39	40–49	2.49 (0.128)	2.67 (0.133)	3 (2, 4)	3 (3, 4)	*χ*² (4) = 23.40	0.001		The 50+ group had significantly higher scores than all other age groups.
		50 and above		3.00 (0.158)		4 (3, 5)					
Attitude	Gender	Male	Female	1.95 (0.084)	1.64 (0.049)	4 (3, 4)	3 (2, 4)	*U* = 132,362	0.004	*r* = −0.11	Males had significantly higher scores (small effect).
	Nationality	Qatari	Non‐Qatari	1.52 (0.055)	2.02 (0.067)	3 (2, 4)	4 (3, 5)	*U* = 137,173	< 0.001	*r* = −0.26	Non‐Qataris had significantly higher scores (medium effect).
	Education	Undergraduate	Postgraduate	1.58 (0.046)	2.35 (0.101)	3 (2, 3)	4 (3, 5)	*U* = 79,848	< 0.001	*r* = −0.34	Postgraduates had significantly higher scores (medium‐to‐large effect).
	Occupation	Full‐time students	Employed	1.54 (0.049)	2.11 (0.079)	3 (2, 4)	4 (3, 5)	*U* = 123,881	< 0.001	*r* = −0.26	Employed individuals had significantly higher scores (medium effect).
	Age	18 and below	19–29	1.53 (0.082)	1.61 (0.057)	3 (2, 4)	3 (2, 4)				
		30–39	40–49	1.86 (0.156)	2.34 (0.156)	3 (2, 4)	4 (3, 5)	*χ*² (4) = 57.67	< 0.001		The 50+ group had significantly higher scores than all other age groups.
		50 and above		2.74 (0.169)		4 (3, 5)					
Practice	Gender	Male	Female	1.66 (0.072)	1.87 (0.043)	3 (2, 4)	3 (2, 4)	*U* = 131,425	0.002	*r* = −0.14	Females had significantly higher scores (small‐to‐medium effect).
	Nationality	Qatari	Non‐Qatari	1.89 (0.051)	1.69 (0.055)	3 (2, 4)	3 (2, 4)	*U* = 158,357	0.024	*r* = −0.05	Qataris had significantly higher scores (small effect).
	Education	Undergraduate	Postgraduate	1.79 (0.042)	1.85 (0.079)	3 (2, 4)	3 (2, 4)	*U* = 106,578	0.292	—	No significant difference
	Occupation	Full‐time students	Employed	1.70 (0.043)	2.02 (0.072)	3 (2, 4)	3 (2, 4)	*U* = 137,179	< 0.001	*r* = −0.15	Employed individuals had significantly higher scores (small‐to‐medium effect).
	Age	18 and below/19–29/30–39/40–49/50+	1.72/1.81/1.90/1.81/1.93		*χ*² (4) = 3.71	0.446		No significant difference			

**Table 4:** Multiple linear regression predicting COVID‐19 practices from attitudes, health habits, and demographic factors.

Predictor	*B*	SE *B*	β	95% CI for *B*	*t*	*p*‐value
(Constant)	0.755	0.089		[0.581, 0.929]	8.49	< 0.001
Attitude score	0.21	0.025	0.21	[0.161, 0.259]	8.41	< 0.001
Health habit score	0.142	0.027	0.142	[0.089, 0.195]	5.25	< 0.001
Female (vs. male)	0.163	0.04	0.163	[0.085, 0.242]	4.05	< 0.001
Qatari (vs. non‐Qatari)	−0.115	0.041	−0.115	[−0.195, −0.035]	−2.83	0.005
Employed (vs. full‐time student)	0.028	0.04	0.028	[−0.050, 0.106]	0.7	0.485

The distribution of COVID‐19 information sources among participants is presented in Figure 1, with social media (66.4%; *n* = 788) and interpersonal communication (54.8%; *n* = 650) being the most used sources.

### 3.3 Multiple Linear Regression Model

To assess the impact of attitude, health habits, and demographic factors on COVID‐19 practice scores, multiple linear regression analysis was conducted. The model included attitude scores and health habit scores as continuous predictors, and sex, nationality, and occupation as categorical predictors. The results are presented in Table 4.

The overall model was statistically significant, *F* (5, 1178) = 75.02, *p* < 0.001, indicating that the predictors collectively explained a significant amount of variance in COVID‐19 practice scores. The adjusted *R*‐squared (*R*²) value of 0.241 indicated that approximately 24.1% of the variance in practice scores could be accounted for by the combination of attitudes, health habits, and the demographic factors included in the model.

Among the predictors, attitude scores (β = 0.210, *p* < 0.001), health habit scores (β = 0.142, *p* < 0.001), and being female (β = 0.163, *p* < 0.001) were positively associated with COVID‐19 practice scores, suggesting that individuals with more positive attitudes, healthier habits, and females were more likely to adhere to recommended practices. Qatari nationality was negatively associated with practice scores (β = −0.115, *p* < 0.001), indicating that Qatari individuals were less likely to follow practices compared to non‐Qataris. Occupation status (employed vs. full‐time student) was not a significant predictor in this model. The positive association observed between female gender and COVID‐19 practice scores should be interpreted with caution. This is particularly important given the predominantly female student population at Qatar University (approximately 77%, as indicated by The Times Higher Education [10]. This demographic imbalance might influence the observed association. Further research into more balanced samples across demographic groups could provide additional clarity.

### 3.4 Comparison of Peak Pandemic and Post‐Pandemic Practices and Behaviors

Table 5 presents the results of the *z*‐tests comparing health practices and behaviors between the peak pandemic and post‐pandemic periods. Statistically significant differences were observed for all practices and behaviors examined. As detailed in Table 5, adherence to preventive measures like social distancing, avoiding crowded gatherings, wearing masks, and hand sanitizer use decreased significantly in the post‐pandemic period compared to peak pandemic levels. However, handwashing frequency remained high post‐pandemic.

**Table 5:** Changes in health practices and behaviors among Qatar university population from peak pandemic to post‐pandemic.

Health practice/behavior	Peak pandemic (%)	Post‐pandemic (%)	*Z‐*score	*p*‐value	Effect size (φ)	Significant change
Attending large gatherings	14.3	46.1	12.13	< 0.001	0.35	↑
Social distancing (1.5 m)	64.8	12.7	−21.46	< 0.001	0.5	↓
Avoiding crowded gatherings	76	18	−22.48	< 0.001	0.52	↓
Wearing a mask regularly	90.5	10.7	−31.14	< 0.001	0.72	↓
Handwashing for at least 20 s	69.9	56.3	−5.11	< 0.001	0.12	↓
Using hand sanitizer regularly	72	36.4	−13.14	< 0.001	0.3	↓
Healthy diet	81.7	49.5	−12.03	< 0.001	0.34	↓
Smoking cessation	14.4	29	6.2	< 0.001	0.14	↑
Taking vitamin supplements	52	43.1	−3.31	0.001	0.08	↓
Regular exercise	61.6	32.4	−10.96	< 0.001	0.26	↓

*Note:* Effect sizes are reported as Phi (φ) for the *z*‐tests of proportions. Phi coefficient is a measure of effect size for the *χ* ^2^ test of association and indicates the strength of association between two categorical variables. The interpretation of the Phi coefficient is as follows:

a. 0.10–small effect.

b. 0.30–medium effect.

c. 0.50–large effect.

Additionally, there were significant changes in health behaviors from peak pandemic knowledge to post‐pandemic habits. The proportion of individuals reporting a healthy diet and regular exercise decreased, while smoking cessation increased significantly. There was also a small, but significant decrease in the use of vitamin supplements.

## 4 Discussion

This study revealed significant shifts in Health Attitudes, Practices, and Sources of Information (HAPS) among Qatar University's population post‐pandemic, highlighting the need for tailored public health interventions.

### 4.1 Demographic Influences on HAPS: A Closer Look

A key finding of this study was the significant variation in HAPS scores across demographic groups, underscoring the need for tailored public health interventions within the university population. Notably, older adults (50+ ; *n* = 69), a group primarily composed of non‐Qatari faculty and staff (approximately 60% based on university data), exhibited the highest median HAPS scores. This aligns with existing literature on age and health consciousness [11, 12, 13], which suggests that older individuals, having accumulated more life experiences and potentially facing increased health risks, may actively seek information and engage in preventive behaviors. Furthermore, this demographic likely has higher education levels and greater exposure to workplace health and safety guidelines.

Disparities were also evident based on nationality. Non‐Qatari participants (*n* = 496) demonstrated significantly higher health habit (median = 3, IQR = 2–5, *p* = 0.015) and attitude (median = 4, IQR = 3–5, *p* < 0.001) scores than Qatari participants (*n* = 690; median = 3, IQR = 2–4 for both), with small to medium effect sizes (*r* = −0.07 and −0.26 respectively). While our data lacks a detailed ethnic breakdown of the non‐Qatari group, it likely includes individuals from various regions, including other Arab countries, South Asia, Southeast Asia, and Western countries. This diverse composition suggests that cultural background, in addition to education and socioeconomic status, may play a crucial role in shaping health behaviors.

Specifically, Qatar's collectivist culture, with its emphasis on group harmony [14], might have initially fostered strong adherence to public health guidelines during the pandemic's peak, particularly among Qatari citizens. The subsequent decline in adherence among Qataris could reflect a shift in the perceived balance between individual liberties and collective responsibility as the immediate threat subsided. Conversely, non‐Qataris, often considered “guests”, might feel a stronger obligation to adhere to local norms, even without strict enforcement. This could be further explained by the “host–guest” dynamic, where non‐Qataris may feel a greater need to conform to local expectations. Additionally, Qatari citizens might feel a greater sense of autonomy in shaping their post‐pandemic behaviors. Differences in risk perception, trust in authority, and communication styles could also contribute to the observed disparities.

These findings highlight the importance of culturally sensitive public health initiatives within the university setting. However, more research, particularly qualitative studies like focus groups or interviews, is needed to fully understand the complex interplay of cultural factors, individual experiences, and health behaviors among the diverse population of Qatar University.

### 4.2 Health Practices and Behaviors: The Pandemic's Dynamic Influence

The COVID‐19 pandemic profoundly influenced health practices and behaviors, as evident in the distinct shifts observed between peak pandemic and post‐pandemic periods (Table 5). During the peak, high adherence to preventive measures was likely driven by heightened risk awareness and strict government regulations. For instance, 90.5% of participants reported regularly wearing a mask during the peak, compared to only 10.7% post‐pandemic. Similar patterns were seen for social distancing and avoiding crowded places, with over 60% adhering to these practices during the peak, plummeting to less than 18% post‐pandemic. This waning adherence can be attributed to a combination of factors, including pandemic fatigue, a decreased perception of risk as the number of new COVID‐19 cases in Qatar significantly dropped from a peak of over 2000 daily cases in May 2020 to less than 100 daily cases in early 2023 [15], and a desire to return to normalcy [16, 17]. Moreover, the Ministry of Public Health in Qatar lifted the mandatory mask mandate in most public spaces in September 2022 [18], further contributing to the observed decrease in mask‐wearing. Public messaging around COVID‐19 also shifted during this period, emphasizing vaccination and living with the virus rather than strict preventive measures. Interestingly, while mask‐wearing and social distancing decreased, handwashing frequency remained relatively high post‐pandemic (56.3%), although it was decreased compared to peak pandemic levels(69.9%). This might be due to the global increase in awareness of hand hygiene during and after the pandemic, while smoking cessation increased post‐pandemic, suggesting that some behaviors promoted during the crisis may have persisted, indicating a positive and potentially lasting impact on hygiene practices. Conversely, the decline in healthy eating and exercise (from 81.7% to 49.5% and from 61.6% to 32.4%, respectively) emphasizes the challenges of maintaining positive behaviors once immediate threats subside, underscoring the need for ongoing reinforcement and support for healthy lifestyle choices [17].

### 4.3 Smoking Cessation: A Public Health Opportunity

The notable increase in smoking cessation intentions post‐pandemic represents a significant public health opportunity. While the effect size (φ = 0.14) may be small, it aligns with global trends observed during the pandemic, suggesting a broader phenomenon of increased awareness about the harmful effects of smoking on respiratory health, particularly in the context of COVID‐19 [19, 20, 21, 22]. This observation is further supported by Shtaiwi et al. [23], who highlighted the potential for COVID‐19 to serve as a catalyst for smoking cessation and relapse prevention. This heightened awareness, coupled with the fear of increased vulnerability to COVID‐19 complications [24], likely contributed to the observed shift towards smoking cessation, which increased from 14.4% to 29%.

Capitalizing on this momentum is crucial for the Ministry of Public Health in Qatar. Implementing targeted interventions, such as widespread anti‐tobacco campaigns, increased access to cessation resources, and personalized support programs, could significantly reduce smoking prevalence and its associated health burdens in the long term.

### 4.4 Information Sources: The Double‐Edged Sword of Social Media

The dominance of social media as the primary source of COVID‐19 information underscores its transformative role in health communication. Its accessibility, speed, and ability to reach diverse audiences make it a powerful tool for disseminating health information and engaging with the public [25, 26]. However, the potential for misinformation and the exaggeration of unverified claims poses significant challenges [27], as demonstrated by Apuke and Omar (2021), who found that fake news related to COVID‐19 was widely shared among social media users [28]. To navigate this landscape effectively, public health officials must actively engage with social media platforms, partnering with trusted influencers and organizations to share accurate, evidence‐based information [29]. Additionally, promoting media literacy and critical thinking skills among the population can empower individuals to discern reliable information from false claims [30].

### 4.5 Multiple Regression: Insights for Targeted Interventions

Our regression analysis further illuminated the factors influencing COVID‐19 practices, revealing that attitude scores (β = 0.210, *p* < 0.001) were a stronger predictor of adherence than health habit scores (β = 0.142, *p* < 0.001), even after accounting for demographic factors. This finding underscores the critical importance of fostering positive attitudes towards public health guidelines, particularly in the context of a pandemic [22]. Public health interventions, therefore, should prioritize strategies that address misconceptions, build trust in health authorities, and resonate with individuals' values and beliefs. Notably, the analysis also revealed a negative association between Qatari nationality and practice adherence (β = −0.115, *p* <0.001). This highlights the need for culturally tailored interventions that consider the unique social and cultural context of the Qatari population, potentially leveraging trusted community leaders, religious figures, and culturally relevant communication channels. The collectivist nature of Qatari society may also play a role, suggesting that interventions should consider how group dynamics influence individual behaviors. However, this study's focus on a predominantly young university population limits the generalizability of these findings to the broader Qatari population. Future research should incorporate more diverse samples, including varied age groups, socioeconomic backgrounds, and geographic locations within Qatar. Additionally, longitudinal designs are needed to track the long‐term sustainability of behavioral changes. While non‐parametric tests were employed in this study due to data distribution, future research with larger, more diverse samples could explore parametric methods after appropriate data checks. By gaining a deeper understanding of the complex interplay between attitudes, habits, demographics, and cultural factors, policymakers and health officials can design more effective strategies to promote adherence to health measures and improve public health outcomes during and after future pandemics.

### 4.6 Limitations and Future Directions

While our study provides valuable insights, it is not without limitations. The sample is confined to the university population in Qatar, predominantly composed of students under the age of 30, limiting the generalizability of our findings to other demographics or the broader Qatari population. Future research should include more diverse samples, potentially utilizing stratified random sampling based on faculty, staff, and student proportions, as well as incorporating participants from different age groups, socioeconomic backgrounds, and geographic locations within Qatar to ensure that interventions are appropriate for the broader population. Additionally, the cross‐sectional nature of our study precludes causal inferences. Longitudinal studies are needed to examine the long‐term application of the newly developed tool HAPS and the sustainability of behavioral changes after the pandemic. Furthermore, the survey was conducted between January and April 2023, approximately 4 months after the lifting of most restrictions in Qatar in late September 2022. This timing is a crucial consideration, as attitudes and practices may have still been in flux during this period. Future studies should consider administering surveys at multiple points post‐restriction lifting to capture the evolving nature of health behaviors. The potential for Type I error due to the disproportionate sample sizes among gender (70.1% female, 29.9% male), education, and employment status groups should also be considered as a limitation of this study. Future research could employ sensitivity analyses or statistical methods specifically designed for imbalanced groups to further examine the robustness of the observed associations. Future studies should also aim for more balanced samples across key demographic variables to provide a more representative understanding of these relationships within the university population. It is important to note that Qatar University's language of instruction is primarily English in most departments, and all candidates are expected to pass English language proficiency requirements [31]. The survey was conducted in English only. While Qatar University's policy requires all candidates to pass English placement tests, future research could explore translating the questionnaire into other relevant languages, such as Arabic, to ensure broader inclusivity and capture a wider range of perspectives. Participants with incomplete responses were excluded from the analysis. Future studies could consider using methods for handling missing data, such as imputation, to assess the potential impact of incomplete responses on the findings.

Additionally, the unequal sample sizes between the KAPS and HAPS studies, while not impacting on demographic comparability, could have limited our ability to detect small but potentially meaningful differences in some comparisons. While the larger HAPS sample provided greater statistical power, the smaller KAPS sample might have reduced our sensitivity to subtle changes. To account for this, we have reported effect sizes alongside *p*‐values in our analysis. These effect sizes quantify the magnitude of the observed differences and provide a more comprehensive understanding of the practical significance of our findings, even in cases where statistical significance might not be reached due to limited power in the smaller sample.

Furthermore, although we employed nonparametric tests due to the ordinal nature of our data, we acknowledge that with our large sample size (*N* = 1186), normality tests are known to be sensitive to even minor deviations from perfectly normal distribution. While nonparametric tests were therefore appropriate for our analysis, future studies will employ additional robustness checks such as Q–Q plots and bootstrapping to further evaluate the distribution of the data and explore the potential application of parametric methods.

In conclusion, this study offers a comprehensive view of the multifaceted impacts of the COVID‐19 pandemic on health behaviors and information sources in Qatar. These findings underscore the need for targeted and adaptable public health interventions, particularly among younger and Qatari populations, to promote sustained healthy habits. Given the evolving nature of public health challenges, interventions for specific behaviors such as mask‐wearing and social distancing should be contextualized within broader public health priorities and the current phase of any given pandemic or health situation. By learning from the pandemic's lessons and capitalizing on positive trends like increased smoking cessation, we can build a more resilient and healthier society in the face of future public health challenges.

## Author Contributions

**Ibrahim Alkaabi:** methodology, investigation, and data curation. **Magdy Abita:** methodology and investigation. **Amr Ouda:** investigation, writing – original draft, methodology, and formal analysis. **Yousif Mahdi:** formal analysis and investigation. **Mohammed Imad Malki:** conceptualization, supervision, investigation, funding acquisition, writing – original draft, writing – review and editing, resources, formal analysis, validation, and data curation.

## Ethics Statement

The studies involving human participants were reviewed and approved by Qatar University Institutional Review Board (QU‐IRB; Number: 1802‐E/23).

## Consent

Written informed consent for participation was not required for this study in accordance with national legislation and institutional requirements.

## Conflicts of Interest

The authors declare that the research was conducted in the absence of any commercial or financial relationships that could be construed as a potential conflict of interest.

## Transparency Statement

The lead author Mohammed Imad Malki affirms that this manuscript is an honest, accurate, and transparent account of the study being reported; that no important aspects of the study have been omitted; and that any discrepancies from the study as planned (and, if relevant, registered) have been explained.

## Supporting information


**Supplementary table 1: The questionnaire. Supplementary Table 2:** A chi‐square test comparison of demographic variables between the HAPS and KAPS populations.

## Data Availability

The data sets used and/or analyzed during the current study are available from the corresponding author upon reasonable request.
