# Supplementary material for: From Knowledge to Habits: Changes in COVID‐19 Health Attitudes, Practices, and Sources of Information (HAPS) at Qatar University Post‐Pandemic
Source: Health Sci Rep. 2025 Jun 4;8(6):e70725. doi: 10.1002/hsr2.70725 (PMC12138042; doi:10.1002/hsr2.70725)
Supplement: Supplementary file 1 — Supplementary table 1: The questionnaire. Supplementary Table 2: A chi‐square test comparison of demographic variables between the HAPS and KAPS populations. [file HSR2-8-e70725-s001.docx]

**Transforming Knowledge into Habits: A Cross-Sectional Comparative Analysis of COVID-19 Health Attitude Practices Sources of information (HAPS) Among Qatar University’s Population**

**Ibrahim Alkaabi^1^, Magdy Abita^2^, Amr Ouda^35^, Yousif Mahdi^4^, Mohammed Imad Malki^3*^**

***Correspondence:**

Dr. Mohammed Imad Malki

momalki@qu.edu.qa

**Supplementary Tables:**

**Supplementary table 1: The questionnaire**

1. Sex

M 🞏 F 🞏

3. Age:

18-28 🞏 29-39 🞏 40-50 🞏 51-60 🞏 61 and above 🞏

4. Nationality:

Qatari🞏 Non-Qatari🞏

5. Educational level:

Undergraduate🞏 postgraduate🞏

6. Occupational status: employed 🞏 unemployed 🞏

**Please indicate if you practice the following:**

| **The first component: Practice** | | **Yes** | **No** | **Sometimes** |
| --- | --- | --- | --- | --- |
| 1 | Do you still attend large social gatherings indoors and outdoors? |  |  |  |
| 2 | Do you still socially distance by at least 1.5 meters from another person? |  |  |  |
| 3 | Do you still avoiding crowded gatherings indoors and outdoors? |  |  |  |
| 4 | Do you still wear a facemask when in public at all times? |  |  |  |
| 5 | Do you still wash your hands with soap and water for at least 20 seconds? |  |  |  |
| 6 | Do you still use hand sanitizer? |  |  |  |

**Have you done the following with regards to COVID-19 laws within the State of Qatar:**

| **The second component: Attitude post COVID-19 laws** | | **Yes** | **No** | **Sometimes** |
| --- | --- | --- | --- | --- |
| 1 | Do you still use the Ehteraz application as set out by the State of Qatar? |  |  |  |
| 2 | Do you still report suspicious symptoms of COVID-19 for yourself, family members, friends or colleagues? |  |  |  |
| 3 | Do you still follow the instructions issued by QU regarding COVID-19 conduct? |  |  |  |
| 4 | Do you still use self-rapid antigen test when having suspicious symptoms of COVID-19? |  |  |  |
| 5 | Do you still self-quarantine when tested positive? |  |  |  |

**Do you still do the followings to boost immunity against COVID-19?**

| **The fourth component: Health Habits post COVID-19 pandemic** | | **Yes** | **No** | **Sometimes** |
| --- | --- | --- | --- | --- |
| 1 | Do you still take vitamins to boost immunity? |  |  |  |
| 2 | Do you still eat healthy food to boost immunity |  |  |  |
| 3 | Do you still exercise regularly to boost immunity? |  |  |  |
| 4 | Do you still consider quit smoking and or passive smoking to boost your immunity? |  |  |  |
| 5 | Do you still vaccinate against sessional infectious diseases? |  |  |  |

**Please indicate which media resources you use to gain information regarding post COVID-19:**

| **The fourth component: Resources** | | **Yes** | **No** | **Sometimes** |
| --- | --- | --- | --- | --- |
| 1 | News channels |  |  |  |
| 2 | Government press conferences |  |  |  |
| 3 | Social Media |  |  |  |
| 4 | Family, relatives, friends and coworkers |  |  |  |

**Supplementary Table 2:** A chi-square test comparison of demographic variables between the HAPS and KAPS populations

| **Chi-Square Tests for age** | | | |  |  |
| --- | --- | --- | --- | --- | --- |
|  | Value | df | Asymptotic Significance (2-sided) |  |  |
| Pearson Chi-Square | 21.873^a^ | 16 | .147 |  |  |
| Likelihood Ratio | 22.235 | 16 | .136 |  |  |
| Linear-by-Linear Association | 4.447 | 1 | .035 |  |  |
| N of Valid Cases | 475 |  |  |  |  |
|  |  |  |  |  |  |
| **Chi-Square Tests for gender** | | | | | |
|  | Value | df | Asymptotic Significance (2-sided) | Exact Sig. (2-sided) | Exact Sig. (1-sided) |
| Pearson Chi-Square | .402^a^ | 1 | .526 |  |  |
| Continuity Correction^b^ | .286 | 1 | .593 |  |  |
| Likelihood Ratio | .401 | 1 | .526 |  |  |
| Fisher's Exact Test |  |  |  | .550 | .296 |
| Linear-by-Linear Association | .402 | 1 | .526 |  |  |
| N of Valid Cases | 475 |  |  |  |  |
|  |  |  |  |  |  |
| **Chi-Square Tests for Nationality** | | | | | |
|  | Value | df | Asymptotic Significance (2-sided) | Exact Sig. (2-sided) | Exact Sig. (1-sided) |
| Pearson Chi-Square | .583^a^ | 1 | .445 |  |  |
| Continuity Correction^b^ | .449 | 1 | .503 |  |  |
| Likelihood Ratio | .583 | 1 | .445 |  |  |
| Fisher's Exact Test |  |  |  | .457 | .251 |
| Linear-by-Linear Association | .582 | 1 | .446 |  |  |
| N of Valid Cases | 475 |  |  |  |  |
|  |  |  |  |  |  |
| **Chi-Square Tests for Educational Level** | | | | | |
|  | Value | df | Asymptotic Significance (2-sided) | Exact Sig. (2-sided) | Exact Sig. (1-sided) |
| Pearson Chi-Square | .866^a^ | 1 | .352 |  |  |
| Continuity Correction^b^ | .693 | 1 | .405 |  |  |
| Likelihood Ratio | .868 | 1 | .351 |  |  |
| Fisher's Exact Test |  |  |  | .376 | .203 |
| Linear-by-Linear Association | .865 | 1 | .352 |  |  |
| N of Valid Cases | 475 |  |  |  |  |
|  |  |  |  |  |  |
| **Chi-Square Tests for Occupational Status** | | | | | |
|  | Value | df | Asymptotic Significance (2-sided) | Exact Sig. (2-sided) | Exact Sig. (1-sided) |
| Pearson Chi-Square | .281^a^ | 1 | .596 |  |  |
| Continuity Correction^b^ | .188 | 1 | .664 |  |  |
| Likelihood Ratio | .281 | 1 | .596 |  |  |
| Fisher's Exact Test |  |  |  | .631 | .332 |
| Linear-by-Linear Association | .280 | 1 | .596 |  |  |
| N of Valid Cases | 475 |  |  |  |  |
